# Beta-Amylase and Phosphatidic Acid Involved in Recalcitrant Seed Germination of Chinese Chestnut

**DOI:** 10.3389/fpls.2022.828270

**Published:** 2022-03-25

**Authors:** Yang Liu, Yu Zhang, Yi Zheng, Xinghua Nie, Yafeng Wang, Wenjie Yu, Shuchai Su, Qingqin Cao, Ling Qin, Yu Xing

**Affiliations:** ^1^Beijing Advanced Innovation Center for Tree Breeding by Molecular Design, College of Plant Science and Technology, Beijing University of Agriculture, Beijing, China; ^2^Key Laboratory for Silviculture and Conservation of Ministry of Education, College of Forestry, Beijing Forestry University, Beijing, China; ^3^Bioinformatics Center, Beijing University of Agriculture, Beijing, China

**Keywords:** SVs, seed germination, recalcitrant seeds, amylase, phosphatidic acid

## Abstract

Chinese chestnut (*Castanea mollissima*), a species with recalcitrant seeds, is an important source of nuts and forest ecosystem services. The germination rate of recalcitrant seeds is low in natural habitats and decreases under conditions of desiccation and low temperature. The germination rate of cultivated Chinese chestnut seeds is significantly higher than that of wild seeds. To explore the reasons for the higher germination rate of cultivated seeds in Chinese chestnut, 113,524 structural variants (SVs) between the wild and cultivated Chinese chestnut genomes were detected through genome comparison. Genotyping these SVs in 60 Chinese chestnut accessions identified allele frequency changes during Chinese chestnut domestication, and some SVs are overlapping genes for controlling seed germination. Transcriptome analysis revealed downregulation of the abscisic acid synthesis genes and upregulation of the beta-amylase synthesis genes in strongly selected genes of cultivated seeds. On the other hand, hormone and enzyme activity assays indicated a decrease in endogenous ABA level and an increase in beta-amylase activity in cultivated seeds. These results shed light on the higher germination rate of cultivated seeds. Moreover, phosphatidic acid synthesis genes are highly expressed in seed germination stages of wild Chinese chestnut and may play a role in recalcitrant seed germination. These findings provide new insight into the regulation of wild seed germination and promote natural regeneration and succession in forest ecosystems.

## Introduction

Seeds act as an important vehicle by which angiosperms to disperse offspring, representing a key stage in the plant life cycle. Seeds are generally classified as “recalcitrant” and “orthodox” based on their tolerance of desiccation (Xia et al., 2014). Recalcitrant seeds die rapidly when stored under desiccation and low-temperature conditions (Walters et al., 2013). Numerous forest species, including *Castanea* (Vieitez et al., 2011), *Quercus* (Sghaier-Hammami et al., 2020), and *Aesculus* (Obroucheva et al., 2016), have recalcitrant seeds. Fagaceae species are the main trees with recalcitrant seeds used to maintain forest ecosystems and play important roles in the survival of wild animals and forest ecological restoration (Walters et al., 2013; Wilf et al., 2019). However, most Fagaceae plant seeds have a low germination rate in the wild, and thus, increasing their germination rate is necessary for afforestation and ecological protection.

To improve the germination rate of recalcitrant seeds, their germination mechanism should be clarified. Several studies have focused on the sensitivity to dehydration and low temperature of recalcitrant seeds in terms of cell biology, physiology and molecular biology (Romero-Rodríguez et al., 2018; Li et al., 2021; Xia and Zhu, 2021), and the findings contribute to the conservation of recalcitrant seeds and the natural regeneration and ecological protection of forests. Seed germination is a complex adaptive trait of higher plants that is influenced by a large number of genes, endogenous hormones and environmental factors (Shu et al., 2016). In species with recalcitrant seeds, high levels of gibberellins (GAs) and low levels of ABA are observed in mature seeds, and the levels of auxin (IAA) and GAs increase during the seed germination process (Romero-Rodríguez et al., 2018). Moreover, exogenous ABA treatments of recalcitrant seeds results in a reduced germination rate (Wang et al., 2019). Genes related to hormone biosynthesis and signal transduction are significantly differentially expressed under desiccation (Dussert et al., 2018; Kijak and Ratajczak, 2020). The *ZEAXANTHIN EPOXIDASE* (*ZEP*), *NINE-CIS-EPOXYCAROTENOID DIOXYGENASE* (*NCED*), *PYRABACTIN RESISTANCE 1* (*PYR1*) genes related to ABA and *YUCCA, ADP-RIBOSYLATION FACTOR* (*ARF*), genes related to IAA have been found to regulate the seed desiccation tolerance acquisition in *Quercus variabilis* (Li et al., 2021). Additionally, paclobutrazol interferes with GA-related gene expression to induce desiccation tolerance in the seeds of *Citrus limon* (Marques et al., 2019). Hormones also regulate seed germination by controlling metabolism, and the amylase activity (Zaynab et al., 2021), lipid metabolism (Cui et al., 2020), glycerophospholipid metabolism (Chen et al., 2018) and reactive oxygen species (ROS) metabolism (Romero-Rodríguez et al., 2018) play key roles in recalcitrant seed germination. Compared to ungerminated seeds, germinated seeds show higher beta-amylase activity (Zaynab et al., 2021). Moreover, there is a close association between reduced seed viability and increasing phosphatidic acid (PA), and glycerophospholipid metabolism genes regulated desiccation sensitivity in recalcitrant seeds (Chen et al., 2018; Li et al., 2021). Nevertheless, a low germination rate of recalcitrant seeds in Fagaceae has not yet been addressed. Most Fagaceae plants grow in the wild, and the reproduction *via* seedlings in afforestation practices mainly relies on seeds. With the cultivation and utilization of forest trees, some cultivated species with high seed germination rates should represent an important source of seedlings for ecological restoration.

Fortunately, there are both wild and cultivated types of Chinese chestnut, which is in the *Castanea* genus. Compared with wild Chinese chestnut seeds, cultivated Chinese chestnut seeds have a higher levels of seed germination capacity under natural conditions. Chinese chestnut (*Castanea mollissima*) has a long history of commercial nut production and is widely cultivated in 26 provinces in China. As starchy nuts, the seeds (dry weight) contain 46∼64% starch, 12∼22% soluble sugars and 0.27∼0.64% lipids (Chen et al., 2017). As the germination characteristics of cultivated seeds are different from those of wild seeds of Chinese chestnut, we explored the mechanisms underlying the seed germination characteristics of Chinese chestnut to provide new insights into recalcitrant seeds.

In this study, we used omics to explore the molecular mechanism of the difference in the germination of recalcitrant seeds of wild and cultivated Chinese chestnut. Recently, wild (HBY_2) and cultivated (N11_1) Chinese chestnut genome assemblies were generated by using Pacific Biosciences single-molecule sequencing technology (Xing et al., 2019; Wang J. et al., 2020), and such Chinese chestnut genomes will provide a platform for comparative genomics analysis to characterize functional and structural features. To identify the differential gene loci related to seed germination of wild and cultivated Chinese chestnut, structural variants (SVs) between wild and cultivated plants were detected through genome comparison and genotyping, and these SV overlapping genes may control seed germination in Chinese chestnut. Comparative transcriptome analysis revealed the related genes involved in the differences in wild and cultivated seed germination. The results help lay a theoretical foundation for improving the recalcitrant seed germination and promoting natural regeneration and succession in forest ecosystems.

## Materials and Methods

### Germination Assay, Endogenous Hormone and Transcriptome Sequencing of Chinese Chestnut Seeds

**FIGURE 1:**
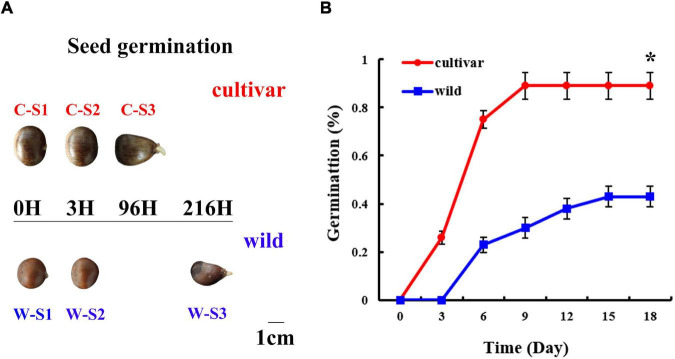
Morphology and physiology of the seed germination process at different developmental stages in wild and cultivated Chinese chestnut. **(A)** Morphology of wild and cultivated Chinese chestnut seeds at different developmental stages of germination. S1: seed embryos after imbibition for 0 h; S2: seed embryos after imbibition for 3 h; S3: radicle emergence at 96 h for cultivated seeds and 216 h for wild seeds. **(B)** The seed germination rate of Chinese chestnut. * indicates a significant difference at *P* < 0.05.

Mature Chinese chestnut seeds were harvested from a wild tree (HBY_2) and a tree of cultivar ‘Jingshuhong’ growing in an orchard in Beijing city, China. Undamaged Chinese chestnut seeds were selected for germination in darkness at 22°C. The seed germination assay included wild and cultivar groups. A total of 270 seeds were equally divided among three biological replicates for each group (Figure 1A). The wild and cultivated seeds were soaked in sterile water for a seed imbibition experiment until the seed weight did not increase. The relative water content was determined as the initial relative water content minus the relative water content that after desiccation. Then, the seeds were planted in a planter (50 cm × 30 cm × 40 cm) containing sterilized sand in a climate chamber with a 16 h light (25°C): 8 h dark (25°C) cycle and 60% relative humidity. The seeds were considered to have successfully germinated when the emerged radicle was at least 5 mm long and the germination rate reached 30% (Roach et al., 2010). According to the germination assay, seed embryos were sampled at 0 (S1) and 3 h of imbibition (S2) and when the seed germination rate reached 30% (S3: 96 h of imbibition for ‘Jingshuhong’ seeds, 216 h for wild seeds), respectively. Three biological replicates were tested for each sample. Endogenous hormone [GA_3_, GA_4_, ABA, IAA, zeatin riboside (ZR), isopentenyladenoside (IPA), brassinosteroids (BR) and methyl jasmonate (MeJA)] contents of these samples were determined by enzyme-linked immunosorbent assays (Deng et al., 2008), and each sample was measured in parallel three times. Alpha-amylase and beta-amylase activities were measured as described previously (Gimbi and Kitabatake, 2002). Then, the RNA of the samples was extracted from the seed embryos for transcriptome sequencing using a Plant RNA Kit (OMEGA, GA, United States), and libraries from the RNA samples were sequenced using the Illumina HiSeq 4000 platform (Illumina, CA, United States) at Novogene Bioinformatics Technology Co., Ltd. (Tianjing, China). Clean data were aligned to the Chinese chestnut V2 and N11_1 genome using STAR (version 2.7.1a). Counts were generated using HTSeq, and DESeq2 was used to identify differentially expressed genes (DEGs).

### Genome Assembly and Chinese Chestnut Genome Annotation

Recalcitrant seed germination is a complex process regulated by various factors. To reveal the basis of the higher seed germination rate of cultivated than wild Chinese chestnut, we used comparative genome analysis to identify related genes. Wild and cultivated genomes of Chinese chestnut have been assembled, but the wild genome still lacks a high-quality chromosomal assembly and annotation version. Therefore, we assembled a chromosome-scale genome of wild Chinese chestnut. Fresh leaf tissue from Chinese chestnut (HBY_2) was used to construct Hi-C libraries, which were sequenced using the Illumina HiSeq PE150 platform (Illumina, CA, United States) at Novogene Bioinformatics Technology Co., Ltd. (Tianjing, China). We utilized Burrows–Wheeler Aligner (BWA) (version 0.7.17-r1188) (Li and Durbin, 2009) to compare the raw Hi-C reads with the draft assembled sequence (Xing et al., 2019); and low-quality reads were removed by SAMtools (version 1.9) (Li et al., 2009). Valid interaction pairs were then applied to build interaction matrices and scale up the major contigs to chromosome-scale scaffolds with LACHESIS (version 201701) (Burton et al., 2013).

For annotation analysis, different tissue types, including leaves, mixed floral buds, leaf buds, stems, female flowers, staminate catkins, nuts, burs, inner and outer shells, globular stage embryos and roots, were collected and immediately frozen in liquid nitrogen. Equal amounts of RNA from these tissues were pooled and used for single-molecule real-time (SMRT) sequencing. Full-length cDNA was synthesized using SMARTer PCR cDNA Synthesis Kit (Clontech Laboratories, CA, United States), and the library was subjected to SMRT sequencing using the PacBio Sequel platform. At the same time, we collected 44 Illumina-based RNA-seq data sets from various tissues, including male flowers, female flowers, leaves, mixed floral buds, male floral buds, nut developmental stages, embryo developmental stages, ovule developmental stages, stems and roots.

Homology alignment and *de novo* annotation were used to identify repetitive elements in the Chinese chestnut genome. For homology-based prediction, the Repbase TE library^1^ was utilized to search against the Chinese chestnut genome using RepeatMasker (version 4.0) (Tarailo-Graovac and Chen, 2009) and RepeatProteinMask^2^ with default parameters. For *de novo* prediction, a *de novo* repetitive element database was constructed by LTR_FINDER^3^, RepeatScout^4^ and RepeatModeler^5^ with default parameters. A hybrid approach of *de novo* prediction, homology-based prediction, and transcriptome-based prediction approaches was applied to identify protein-coding regions and to predict genes. For homology-based prediction, Exonerate (version 2.47.3) (Slater and Birney, 2005) was employed for mapping against *C. mollissima*, with protein sequences from *Arabidopsis thaliana*, *Oryza sativa*, *Vitis vinifera*, *Populus trichocarpa*, *Malus domestica*, *Cucumis sativus*, *Juglans microcarpa* × *Juglans regia*, *Casuarina equisetifolia* and *Quercus robur*. Homologous proteins were then aligned to the Chinese chestnut genome by TBLASTN (Altschul et al., 1997), which were then aligned to the assembly by GeneWise (v2.4.1) (Birney et al., 2004) to predict gene structures. For *de novo* prediction, Augustus (version 3.0.2) (Stanke et al., 2006), GlimmerHMM (version 3.0.2) (Majoros et al., 2004), SNAP (version 11-29-2013) (Korf, 2004), GeneID (version 1.4) (Parra et al., 2000), and GENSCAN (version 1.0) (Salamov and Solovyev, 2000) were used to predict coding regions in the repeat-masked genome. For transcriptome-based prediction, Cufflinks (version 2.1.1) (Trapnell et al., 2012) was used to link transcripts from the TopHat results to gene models, and then PASA (version 2.3.3) (Campbell et al., 2006) was applied on the basis of the assembled RNA-seq unigenes. Finally, an integrated gene set from the above three methods was produced by EVidenceModeler (EVM version 1.1.1) (Haas et al., 2008).

### Gene Functional Annotation

In a previous study, a standard pipeline was established for thoroughly annotating predicted protein-coding genes (Zheng et al., 2019). In brief, the predicted gene sequences were compared to the *Arabidopsis* protein (TAIR), NCBI non-redundant (nr), and UniProt (Swiss-Prot and TrEMBL) databases using the BLASTP command of DIAMOND (Buchfink et al., 2015), with an *E*-value cutoff of 1e^–5^. Furthermore, InterProScan was used to identify functional domains in all protein sequences by comparing them to the InterPro database (Mitchell et al., 2019). To assign GO terms for each protein-coding gene, the BLASTP results from the nr database and the discovered InterPro domains were input into the Blast2GO pipeline (Conesa and Götz, 2008). The BLASTP leads obtained from searches against the TAIR and UniProt databases were input into the AHRD program^6^ to acquire accurate, succinct and useful gene functional summaries. The KEGG pathways encoded by each of the *Castanea* genomes were also predicted by KEGG Automatic Annotation Server^7^. The iTAK program was employed to identify TFs and TRs from the predicted protein-coding genes and to classify them into different families (Zheng et al., 2016).

### Comparative Genomic Analysis and Detection of Structural Variants Between the V2 and N11_1 Genome

Changes in SV allele frequency across distinct Chinese chestnut populations are a consequence of domestication processes such as favorable trait selection and introgression from ancestral groups. We analyzed SV allele frequency changes from wild to cultivated materials for domestication to discover SVs under selection during Chinese chestnut domestication and breeding. The relation pipelines for SV detection and genotyping in Chinese chestnut were based on the study of Wang X. et al. (2020). SVs of Chinese chestnut genomes V2 and N11_1 were identified by Minimap with the parameter “-ax asm5” (Li, 2018). The resulting alignments were analyzed using Assemblytics (v1.1) (Nattestad and Schatz, 2016) for SV identification. SV calling was based on genome comparison, and then filtering by Illumina read mapping.

### Structural Variants and SNP Genotyping in the Chinese Chestnut Population

A total of 60 accessions were collected, including 28 wild and 32 cultivated samples of Chinese chestnut in China (Supplementary Data 1). Young leaves were immediately frozen in liquid nitrogen and stored at −80°C; genomic DNA was extracted from young leaves using the DNeasy Plant Mini Kit (Qiagen, Hilden, Germany) and used to construct sequencing libraries. Paired-end sequencing libraries with an insert size of approximately 450 bp and a read length of 150 bp were sequenced using an Illumina HiSeq 2500 sequencer. The raw reads were filtered to obtain high-quality sequences by Trimmomatic (v0.39) (Bolger et al., 2014). The high-quality reference SVs identified between the V2 and N11_1 genome were genotyped in the 60 Chinese chestnut accessions based on the protocols of Gao et al. (2019) and Wang X. et al. (2020). Clean Illumina reads for each accession were aligned to the V2 and N11_1 genome by BWA-MEM (v0.7.17) (Li and Durbin, 2009), with no more than 3% mismatches allowed. SVs in each accession were classified as V2 (same as V2), N11_1 (same as N11_1), and heterozygous (carrying both V2 and N11_1 alleles) genotypes. The allele frequency of certain SVs was computed for each group, and the significance of the frequency differences between two compared groups was established using Fisher’s exact test. Raw *P* values of SVs were adjusted using the false discovery rate (FDR) approach, and SVs with corrected *P* values < 0.05 were considered as those under selection.

In addition, we employed Illumina reads matched to the V2 genome for SNP calling. Variant calling was performed independently with Sentieon (Freed et al., 2017) with default parameters. GATK (v4.1.1.0) was used for hard filtering with the settings parameters “QD < 2.0 || FS > 60.0 || MQ < 40.0 || MQRankSum < −12.5 || ReadPosRankSum < −8.0” (McKenna et al., 2010). SNPs with at least 70% genotyped accessions, with a minor allele frequency (MAF) of no less than 0.03 and overlapping with known SNPs from the V2 and N11_1 genome alignment were preserved.

### Population Genetic Analysis

IQ-TREE (v2.0.3) with maximum likelihood was used to build phylogenetic trees for the Chinese chestnut accessions using the full SNPs at fourfold degenerated sites (4DTV) in 1,000 bootstrap runs with *Castanea henryi* as the outgroup (Sun et al., 2020). Principal component analysis (PCA) was performed using the program Plink (v1.90) (Purcell et al., 2007). VCFtools (Danecek et al., 2011) was used to calculate *F*_ST_ and π across the genome with a 15 kb window based on the SNPs. XP-CLR analyses were implemented to detect selective sweeps based on SNPs across chestnut populations (Chen et al., 2010). Correlation coefficients (*r*^2^) were calculated for all pairs of SNPs to measure linkage disequilibrium (LD) decay using PopLDdecay (v3.41) (Zhang et al., 2019) with default parameters.

## Results

### Physiological Features of Seed Germination in Wild and Cultivated Chinese Chestnut

As based on observation of the seed germination process, 0–12 h (0–12H) represented a rapid initial penetration phase; and 12–48 h (12–48H) represented a plateau phase in terms of water content (Supplementary Figure 1). There was a significant difference in the water content of seeds, with averages water contents of 46 and 52% for wild and cultivated Chinese chestnut seeds before the water uptake phase, respectively (Supplementary Figure 1). Radicles started to emerge after imbibition for 96 h (4 days) and 216 h (9 days) in wild and cultivated Chinese chestnut seeds, respectively (Figure 1A). There were significant changes in the seed germination rate, with total germination rates of 43 and 89% for wild and cultivated Chinese chestnut seeds, respectively (Figure 1B). The cultivated and wild seed germination rates reached a maximum at 9 and 15 days, respectively.

### Genomic Structural Variants Between Wild and Cultivated Genomes

**FIGURE 2:**
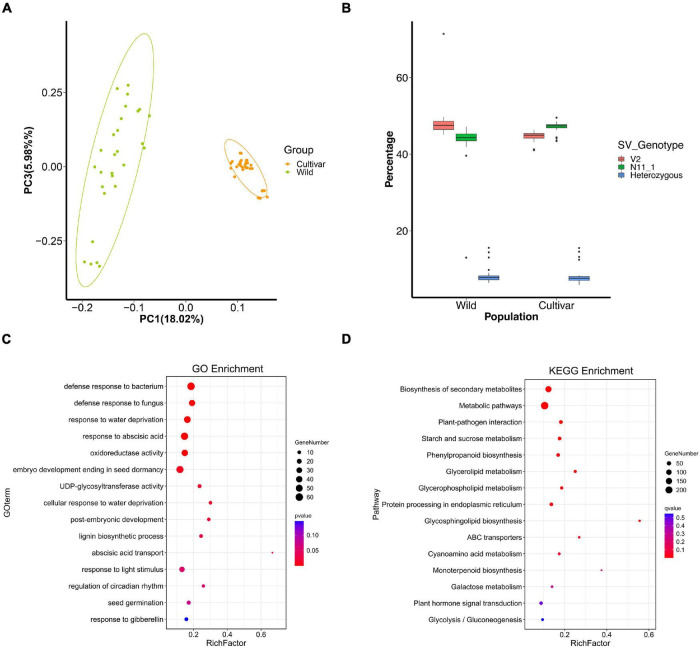
SVs under selection during Chinese chestnut domestication and breeding. **(A)** PCA plots of wild and cultivated Chinese chestnut accessions. **(B)** Percentages of SVs with genotypes in different populations. **(C)** GO enrichment of genes with strongly selected SVs in Chinese chestnut. **(D)** KEGG enrichment of genes with strongly selected SVs in Chinese chestnut.

We assembled a chromosome-scale genome of wild Chinese chestnut, and a total of 2,652,199 read pairs (90.55 Gb clean reads) were acquired by Hi-C sequencing, offering over 115-fold coverage (Supplementary Tables 1, 2). Then, to improve gene prediction accuracy, high-quality transcripts of 11 mixed tissue types were sequenced using a single-molecule real-time sequencer from Pacific Biosciences (Supplementary Table 3). Finally, a total of 33,991 protein-coding genes were aligned to the wild Chinese chestnut genome (V2) (Supplementary Figure 2 and Supplementary Tables 4, 5). The Chinese chestnut V2 genome assembly can be accessed at *Castanea* Genome Database (CGD^8^). Indels were discovered through direct comparison of the genomes of wild (V2) and cultivated (N11_1) Chinese chestnut with mapping of PacBio long reads. A total of 113,524 indels were detected, ranging in size from 10 to 20,094 bp, including intergenic, intronic, upstream, downstream, and exonic areas (Supplementary Data 2 and Supplementary Table 6). Most of the indels are short, with 87.7% being less than 100 bp and only 0.6% exceeding 10 kb (Supplementary Figure 3). To further identify the SVs under strong environmental and artificial selection, 113,524 indels were genotyped in the Chinese chestnut population to investigate allele frequency changes, including 28 wild and 32 cultivated accessions (Supplementary Data 1). The short read sequencing data yielded 796 Gb of high-quality clean data, and the sequencing depths ranged from 10× to 42× in the wild and cultivated populations of Chinese chestnut. Genotyping the SVs in two reference genomes using Illumina short reads further supported their high specificity (Supplementary Table 6). Phylogenetic analysis and PCA clearly separated the wild and cultivated groups of Chinese chestnut (Figure 2A and Supplementary Figure 4). LD values were also calculated among wild and cultivated populations, and higher LD was found in the cultivated population (Supplementary Figure 5).

### Selection of Structural Variants for Seed Germination

**FIGURE 3:**
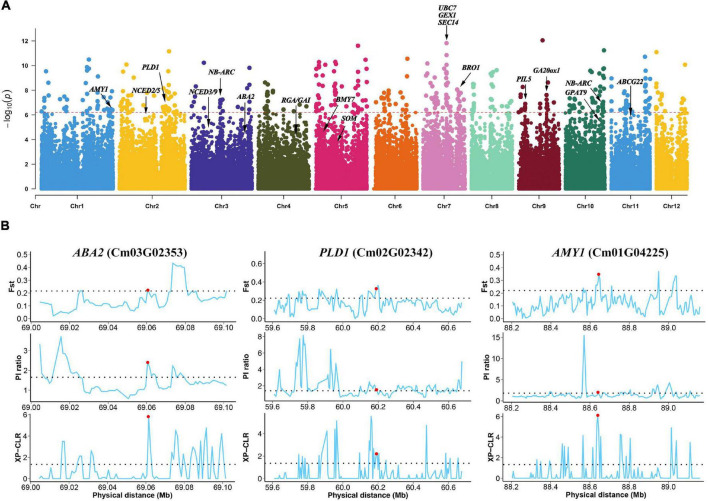
Strong selection of genes between wild and cultivated Chinese chestnut. **(A)** Genome-wide distribution of selective sweeps in Chinese chestnut. **(B)** *F*_ST_, π, and XP-CLR values across the genomic regions of the *ABA2*, *PLD1* and *AMY1* genes. The dashed horizontal line represents the selection threshold (top 5% of the genome). Red dots denote the genes that are connected.

Structural variant loci with the V2 alleles were common in the wild population, accounting for 48.46% of the SVs in each accession, whereas 43.48% of SV loci showed the homozygous N11_1 genotype (Figure 2B). The allele frequencies of 4,892 SVs were significantly differentially changed between the wild and cultivated populations of Chinese chestnut (Supplementary Data 4), and these indels might affect 4,673 genes in the V2 genome. These genes are significantly involved in biosynthesis of secondary metabolites, plant-pathogen interaction, starch and sucrose metabolism, and glycerophospholipid metabolism according to KEGG enrichment function (Figure 2D), and response to abscisic acid, response to water deprivation, and seed germination according to GO enrichment function (Figure 2C and Supplementary Data 3, 5). These genes were also selected at the sequence level using *F*_ST_, nucleotide diversity (π), and the cross-population composite likelihood ratio (XP-CLR) (Figure 3).

Interestingly, some genes involved in plant hormone signal transduction and starch and sucrose metabolism were detected, and these genes may influence the differences in seed germination between wild and cultivated Chinese chestnut. The regions included a 12 bp insertion in the exon of *PHYTOCHROME INTERACTING FACTOR 3-LIKE 5* (*PIL5*, Cm09G00427), and an 18 bp deletion in the intergenic region of *SOMNU*S (*SOM*, Cm05G01513), *ABA DEFICIENT 2* (*ABA2*, Cm03G02353) for ABA biosynthesis and *GIBBERELLIN 20 OXIDASE 1* (*GA20ox1*, Cm09G01563) for GA biosynthesis in plant hormone signal transduction and *ALPHA-AMYLASE-LIKE 1* (*AMY1*, Cm01G04225) and *BETA-AMYLASE 7* (*BMY7*, Cm05G00315) for starch degradation *via* alpha-amylase and beta-amylase activities (Figure 3B). Additionally, some genes involved in glycerophospholipid metabolism were detected in wild and cultivated Chinese chestnut, including a 17 bp deletion in phospholipase D (*PLD1*, Cm02G02342) and a 39 bp deletion in *GLYCEROL-3-PHOSPHATE ACYLTRANSFERASE 9* (*GPAT9*, Cm10G02185), which are involved in activating the synthesis of phosphatidic acid. The *ARABIDOPSIS THALIANA ATP-BINDING CASSETTE G22* (*ABCG22*, Cm11G01020) gene, an ABC transporter gene, is involved in drought susceptibility in seed germination. Moreover, the 1.61 Mb genomic region with a high selection score contains four genes on chromosome 7 (Figure 3A), including *UBIQUITIN CARRIER PROTEIN 7* (*UBC7*, Cm07G01553) and *SECRETION 14* (*SEC14*, Cm07G01557), which are involved in plant responses to multiple stress conditions (Ascencio-Ibáñez et al., 2008; Feng et al., 2020). *GAMETE EXPRESSED PROTEIN 1* (*GEX1*, Cm07G01555) contributes to gametophyte development (Alandete-Saez et al., 2011), suggesting strong selection of this region during domestication. In addition, there are several regions of genes involved in plant pathogen interactions, including a 33 bp deletion in the promoter of *NB-ARC* (Cm10G02154) and a 17 bp deletion in *NB-ARC* (Cm03G01169), were detected. These genes may contribute to differences in disease resistance and responses to stress conditions between wild and cultivated Chinese chestnut. The results demonstrated a common selection preference for the N11_1 allele in Chinese chestnut domestication and improvement.

### Selected Structural Variants Affecting Expression of Seed Germination Genes in Wild and Cultivated Chinese Chestnut

To further verify the strongly artificially selected genes and identify DEGs associated with seed germination in wild and cultivated Chinese chestnut, stage S1–S3 samples were used for transcriptomic sequencing analysis based on seed germination phases. There were 5,562, 6,741 and 9,105 DEGs in stages S1, S2 and S3 of cultivated Chinese chestnut seed germination, respectively. According to KEGG pathway analysis, these DEGs are significantly associated with the biosynthesis of secondary metabolites, metabolic pathways, starch and sucrose metabolism, and plant hormone signal transduction (Supplementary Figure 6). These genes may be related to recalcitrant seed germination in Chinese chestnut.

**FIGURE 4:**
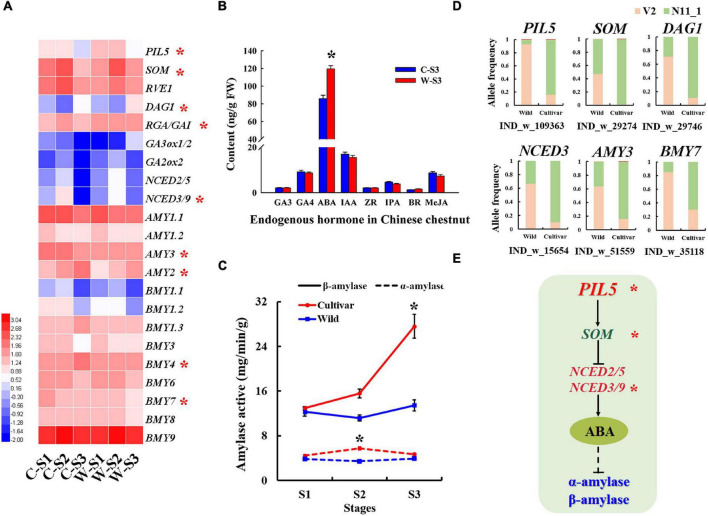
Selected amylase genes associated with the germination of Chinese chestnut seeds. **(A)** Seed germination-related gene expression profiles of Chinese chestnut, where * indicates a significant difference at *P* < 0.05. **(B)** Levels of endogenous hormones in Chinese chestnut at seed germination stage S3, where * indicates a significant difference at *P* < 0.05. **(C)** Alpha-amylase and beta-amylase activities in seed germination stages of wild and cultivated Chinese chestnut seeds; * indicates a significant difference at *P* < 0.05. **(D)** The allele frequencies of selected SVs in wild and cultivated Chinese chestnut. **(E)** The model of seed germination regulation by ABA synthesis pathway genes under strong selection in Chinese chestnut; * indicates the significance of the key genes.

In general, seed germination was influenced by alpha-amylase and beta-amylase activity. Notably, the *AMY* and *BMY* genes exhibited allele frequency change patterns indicative of strong selection in the wild and cultivated populations of Chinese chestnut. For example, a 599 bp deletion in the *AMY3* gene promoter had a frequency of 63.64% in the wild population and 16.13% in the cultivated population in the V2 genome, and the 11 bp deletion in the *BMY7* gene had a frequency of 85.19% in the wild population and 30.00% in the cultivated population in the V2 genome (Figure 4D). Furthermore, the *AMY1*, *AMY2*, and *AMY3* genes were significantly more highly expressed during the S1–S2 stages of cultivated seed germination (Figure 4A) and may be involved in the differential of the alpha-amylase activity in the seed imbibition stage of wild and cultivated Chinese chestnut (Figure 4C). In the radicle emergence stage (S3), the beta-amylase activity of cultivated seeds was significantly higher than that of wild seeds, and there was no significant difference in alpha-amylase activity between cultivar and wild seeds (Figure 4C). The *BMY4* and *BMY7* genes were strongly selected in the wild and cultivar genomes and significantly highly expressed in the radicle emergence stage (S3) of the cultivated seeds; it may be involved in the differential of beta-amylase activity between of wild and cultivated seeds in the S3 stage (Figures 4A,C). Additionally, according to the SV divergence across the cultivated and wild genomes, GA and ABA signal transduction genes may be involved in seed germination differences. The *NCED2/5* and *NCED3/9* genes were strongly selected and showed specific allele frequency change patterns (Figure 4D). We also found that several genes of the ABA metabolic pathway to be significantly upregulated expression in stage S3 of wild Chinese chestnut, including *ABA4*, *ABA2*, *NCED2/5*, *NCED3/9* and *ABSCISIC ALDEHYDE OXIDASE 3* (*AAO3*) (Supplementary Figure 8). In particular, *NCED2/5* and *NCED3/9* were activated in wild seed germination stage S3. The *PIL5* gene was also detected in the SV divergence analysis of the genome and may be involved in seed germination. In terms of gene expression, the *PIL5* gene was significantly more highly expressed in wild seed germination stages, which directly activates transcription of the *SOM*, GIBBERELLIC ACID INSENSITIVE*/REPRESSOR OF GA* (*GAI/RGA*) and *DOF AFFECTING GERMINATION 1* (*DAG1*) genes to regulate the expression of genes related to GA and ABA biosynthesis, and was significantly more highly expressed in the wild seed germination stages (Supplementary Figure 9). Moreover, expression of the GA biosynthetic genes *GA3ox1/GA3ox2* was suppressed in stages S1–S2 and activated in stage S3 of wild seed germination, and *GA2ox2* was activated in wild seed germination stages S1–S3 (Figure 4A and Supplementary Figure 7). In addition, the content of the endogenous hormone ABA was significantly higher in wild seeds than in cultivated seeds (Figure 4B), consistent with agreement with the role of *NCED2/5* and *NCED3/9* in the activation of this direct regulation of ABA levels. There were no significant changes in the contents of other endogenous hormone in the cultivated and wild seed germination stages (Figure 4B).

**FIGURE 5:**
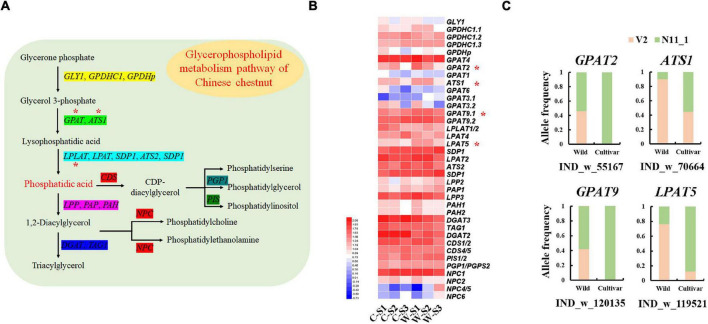
Glycerophospholipid metabolism genes associated with the germination of Chinese chestnut seeds. **(A)** Model of the glycerophospholipid metabolism pathway in Chinese chestnut; * indicates significant key genes. **(B)** The glycerophospholipid metabolism gene expression profiles of Chinese chestnut, where * indicates a significant difference at *P* < 0.05. **(C)** Allele frequencies of selected SVs in wild and cultivated Chinese chestnut.

Although the lipid content of Chinese chestnut seeds was less than 1%, we found that glycerophospholipid metabolism genes were strongly selected in the wild and cultivated populations (Figure 2D). The *GLYCEROL-3-PHOSPHATE SN-2-ACYLTRANSFERASE 2* (*GPAT2*), *GPAT9*, *ACYLTRANSFERASE 1* (*ATS1*) and *LYSOPHOSPHATIDYL ACYLTRANSFERASE 5* (*LPAT5*) genes showed specific allele frequency change patterns. For example, a 45 bp deletion in the *LPAT9* gene had a frequency of 76.19% in the wild group and 12.50% in the cultivated group (Figure 5C). These genes, which are involved in the synthesis of phosphatidic acid (Figure 5A), were significantly highly expressed during wild seed germination (Figure 5B). Additionally, the other genes involved in phosphatidic acid synthesis showed high expression during wild seed germination, although they were not strongly selected in the genomes.

## Discussion

Seed germination is crucial for plant development and breeding programs. This process is affected by environmental conditions and genetic structure, and plants require large amounts of energy, such as starch, proteins, and lipids during germination (Zhao et al., 2018; Zaynab et al., 2021). The starchy nuts of Chinese chestnut have a dry weight of 46∼64% (Chen et al., 2017), and the germination characteristics of these recalcitrant seeds differ between cultivated and wild trees. In general, seeds are unable to survive drying and chilling, as they rapidly lose their germination capacity and viability during storage (Pammenter and Berjak, 1999), and wild seeds have a strong dormancy capacity and low germination rates (Nakamura et al., 2017; Wang et al., 2018). Various factors are responsible for the low rate of seed germination, among which amylase has been recognized as an important factor in regulating seed germination (Damaris et al., 2019). In the present study, cultivated seeds displayed a stronger germination capacity than wild seeds. Some strongly selected alpha-amylase and beta-amylase genes were identified by comparing the genomes V2 and N11_1. *BMY4* and *BMY7* were significantly more highly expressed during the germination stages of cultivated seeds, and the activity of beta-amylase was significantly higher than that of alpha-amylase during the seed germination stage. These findings implied that beta-amylase may be play a key role in the high rate of seed germination processes of cultivated Chinese chestnut. Similarly, beta-amylase activity has been also verified to be an important factor in recalcitrant seed germination (Zaynab et al., 2021). Moreover, the amylase gene encodes an enzyme involved in starch degradation and is regulated by ABA and GA (Vanstraelen and Benková, 2012). ABA biosynthetic genes were strongly selected, with suppressed expression to decrease ABA levels in cultivated Chinese chestnut seeds. *PIL5*, a key negative regulator of seed germination, activates the expression of *SOM* by binding directly to its promoter, partially regulating expression of ABA and GA metabolic genes (Oh et al., 2004; Kim et al., 2008). Among the regulatory genes associated with Chinese chestnut seed germination (Figure 4E), high expression of the *PIL5* gene is also involved in the differential germination between wild and cultivated seeds.

Lipid degradation and conversion to sugars are the key factors for seed germination in oil palm (Cui et al., 2020). Membrane lipid analysis in relation to recalcitrant seed desiccation tolerance has a close relationship with a reduced seed germination rate (Chen et al., 2018), and phosphatidic acid synthesis genes are involved in the dehydration sensitivity of recalcitrant seed germination in cork oak (Li et al., 2021). Moreover, phosphatidic acid results in a response to ABA during seed germination (Katagiri et al., 2005). Regardless, it remains unclear whether the lipids are involved in the germination of starchy seed species. Although the lipid content of Chinese chestnut is only 0.27∼0.64% (Chen et al., 2017), there are some strongly selected genes involved in phospholipid metabolism and phosphatidic acid synthesis in cultivated and wild genomes, and these genes were upregulated in wild seed germination stages. This indicates that phospholipids may be involved in the germination of Chinese chestnut seeds.

**FIGURE 6:**
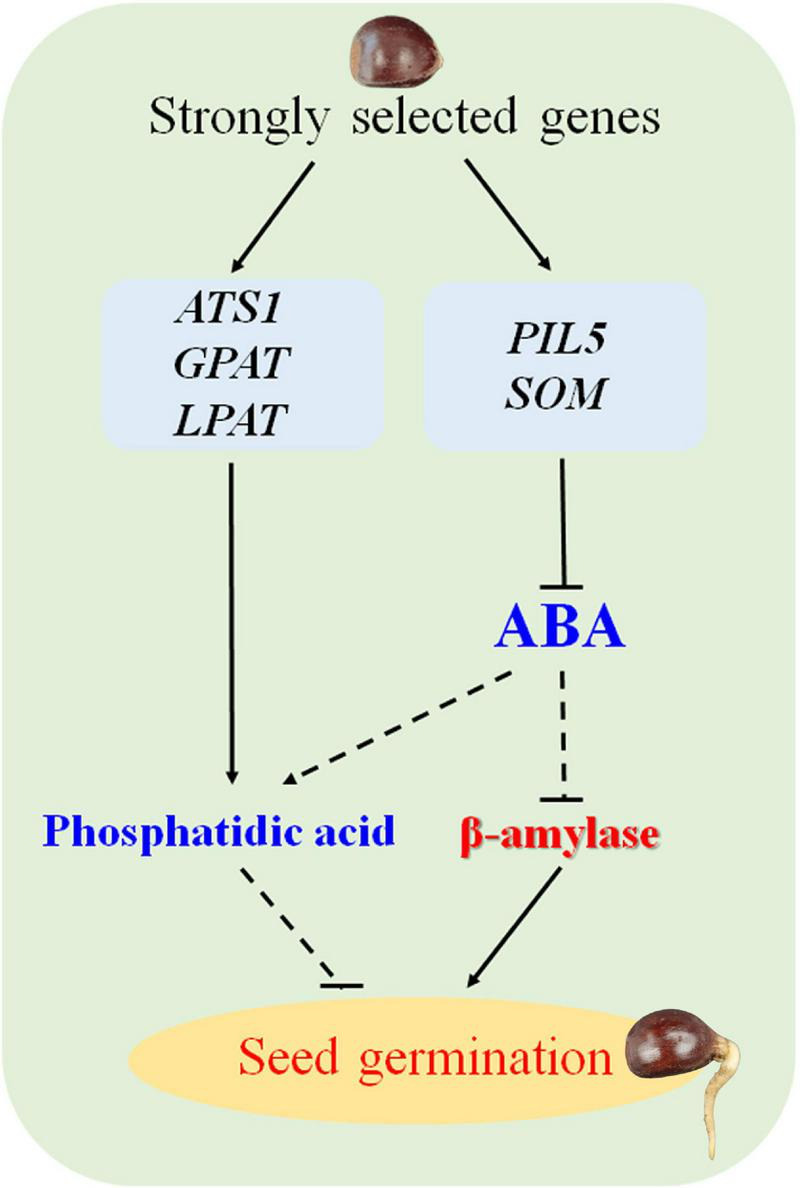
Model of the seed germination mechanism regulated by strongly selected genes in Chinese chestnut.

The germination rate of the highly recalcitrant seeds of cultivated the Chinese chestnut was influenced by several factors, including natural and artificial selection. Finally, a possible working model for proposed for the regulatory mechanism of germination in recalcitrant seeds of Chinese chestnut is proposed (Figure 6). These findings will contribute to improving the germination rate of recalcitrant seeds and provide insight into recalcitrant seed germination.

## Data Availability Statement

The sequencing datasets presented in this study can be found in online repositories. The Chinese chestnut genome assembly can be accessed at Castanea Genome Database (www.castaneadb.net). The names of the repository/repositories and accession number(s) can be found in the article/Supplementary Material.

## Author Contributions

YX and LQ designed the project. YL and YX wrote and revised the manuscript. YuZ, YiZ, and YL analyzed the data and constructed the database. YL, YuZ, YW, WY, and XN collected the samples. SS and QC participated in the manuscript. All authors revised and approved the manuscript.

## Conflict of Interest

The authors declare that the research was conducted in the absence of any commercial or financial relationships that could be construed as a potential conflict of interest.

## Publisher’s Note

All claims expressed in this article are solely those of the authors and do not necessarily represent those of their affiliated organizations, or those of the publisher, the editors and the reviewers. Any product that may be evaluated in this article, or claim that may be made by its manufacturer, is not guaranteed or endorsed by the publisher.

## Footnotes

1
http://www.girinst.org/repbase/

2
http://www.repeatmasker.org/

3
http://tlife.fudan.edu.cn/ltr_finder/

4
http://www.repeatmasker.org/

5
http://www.repeatmasker.org/RepeatModeler.html

6
https://github.com/groupschoof/AHRD

7
https://www.genome.jp/tools/kaas/

8
www.castaneadb.net

## Supplementary Material

Supplementary Data 4 GO and KEGG enrichment results of were strongly selected genes.

Supplementary Data 5 The allele frequencies of SVs in Chinese chestnut.
