# Supplementary material for: Beta-Amylase and Phosphatidic Acid Involved in Recalcitrant Seed Germination of Chinese Chestnut
Source: Front Plant Sci. 2022 Mar 25;13:828270. doi: 10.3389/fpls.2022.828270 (PMC8990265; doi:10.3389/fpls.2022.828270)
Supplement: Supplementary file 1 [file Data_Sheet_1.DOCX]

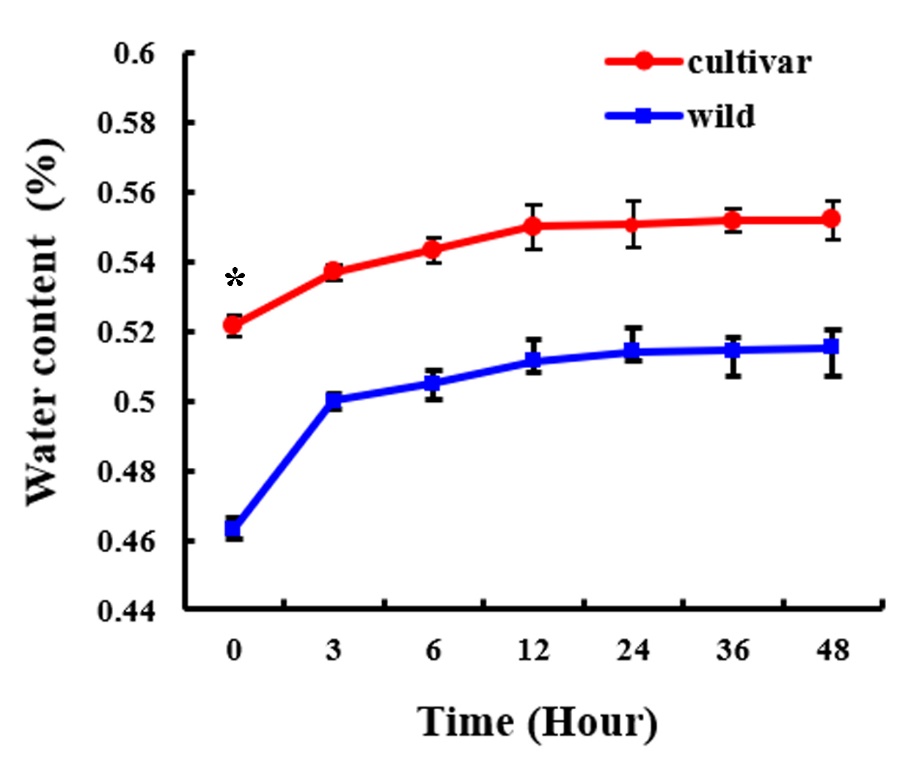


Figure S1 Water content of wild and cultivated Chinese chestnut seeds.

* indicates a significant difference at *P* < 0.05.


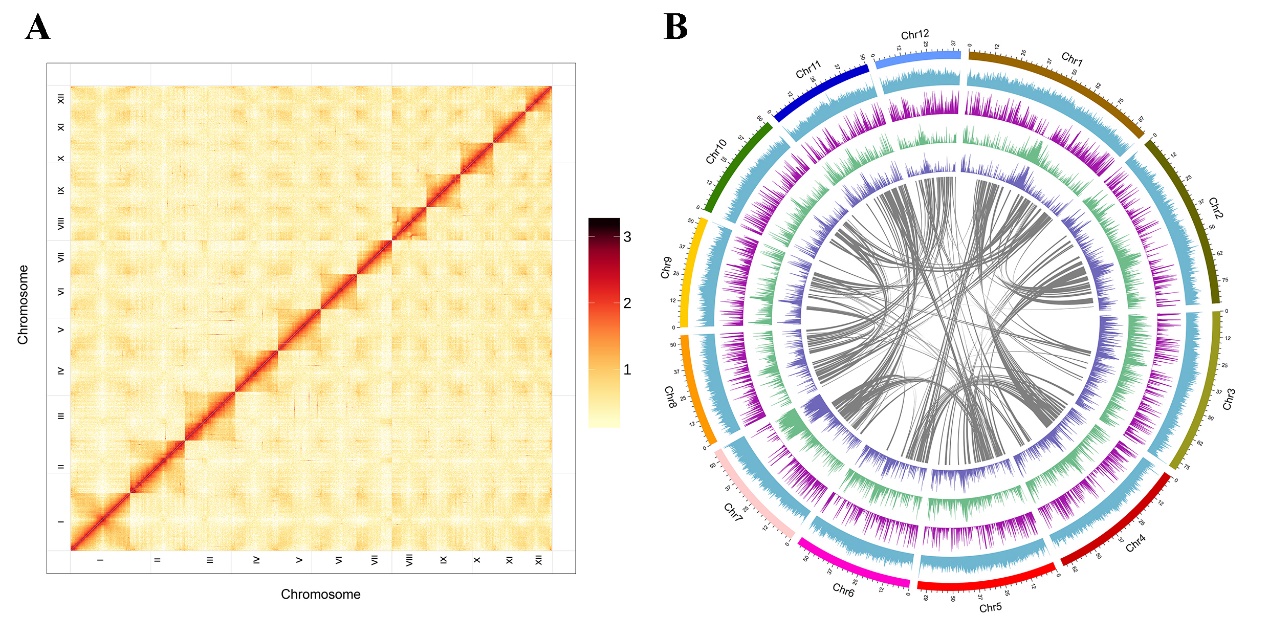


Figure S2 Characterization of the Chinese chestnut V2 genome.

(A) Hi-C interaction heat map for the Chinese chestnut V2 genome. (B) Features of the Chinese chestnut V2 genome. The tracks from outside to inside represent the 12 pseudomolecules with anchored contigs, GC content, gene density, repetitive sequence density, LTR retrotransposon density, and syntenic blocks.


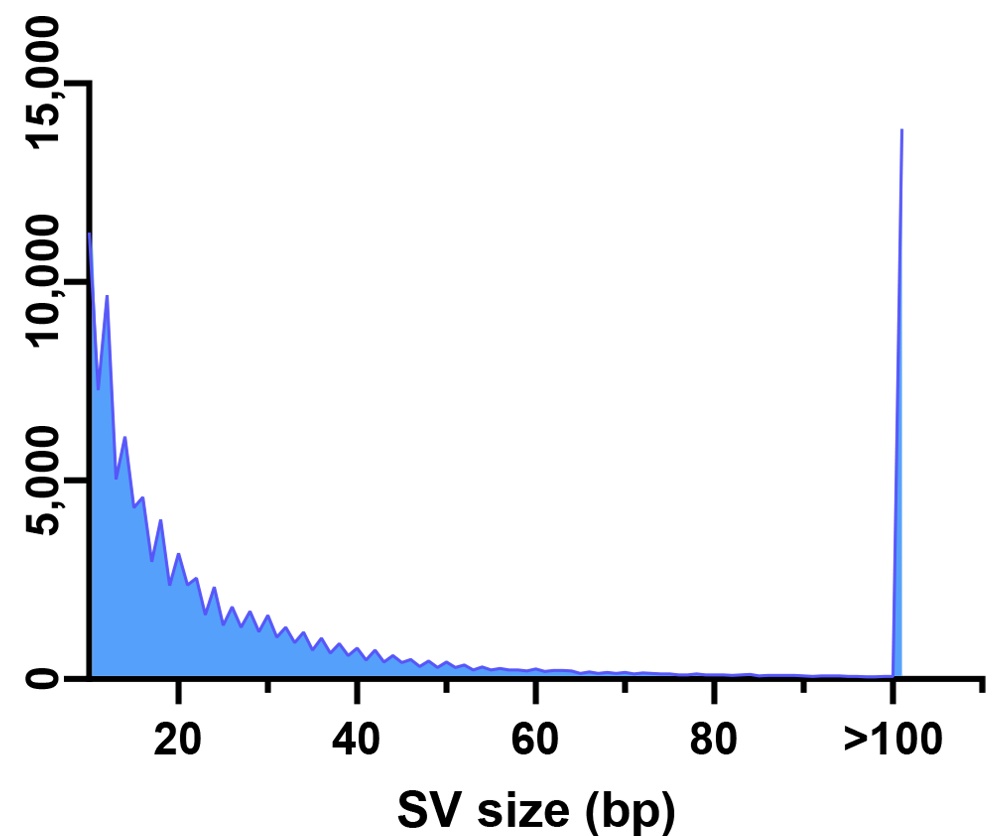


Figure S3 Distribution of SV sizes between the two Chinese chestnut V2 and N11_1 genome.


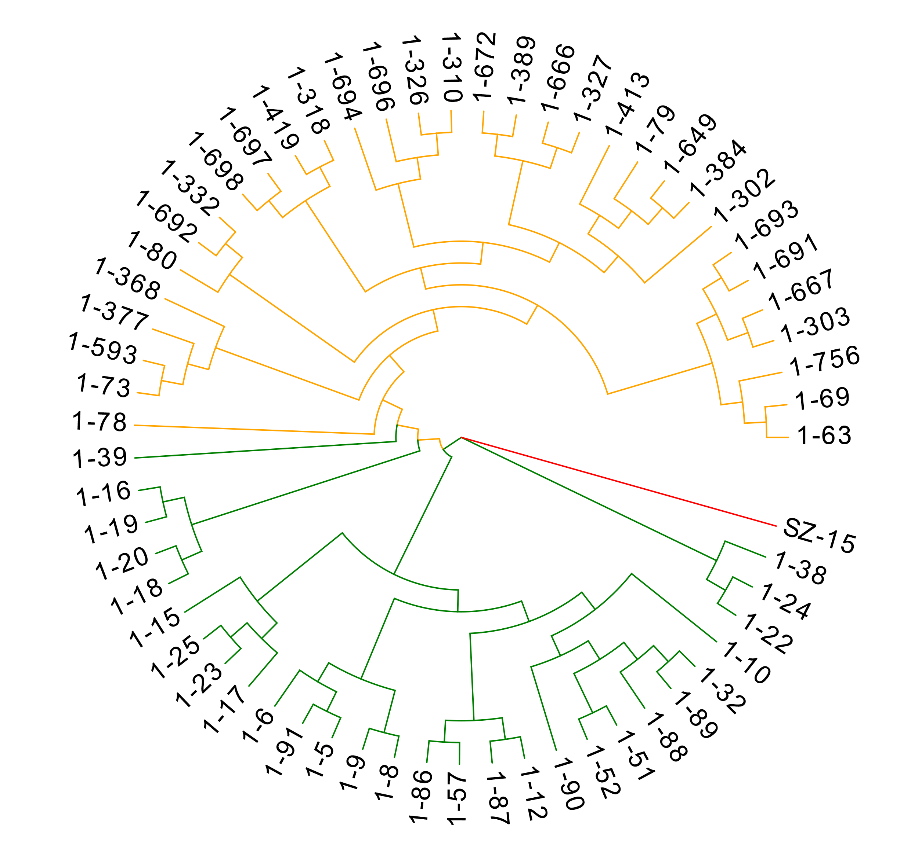


Figure S4 Phylogenetic tree of the wild and cultivar Chinese chestnut accessions.

SZ-15 (a *Castanea henryi* accession) as the outgroup for the phylogenetic tree (Sun et al., 2020)


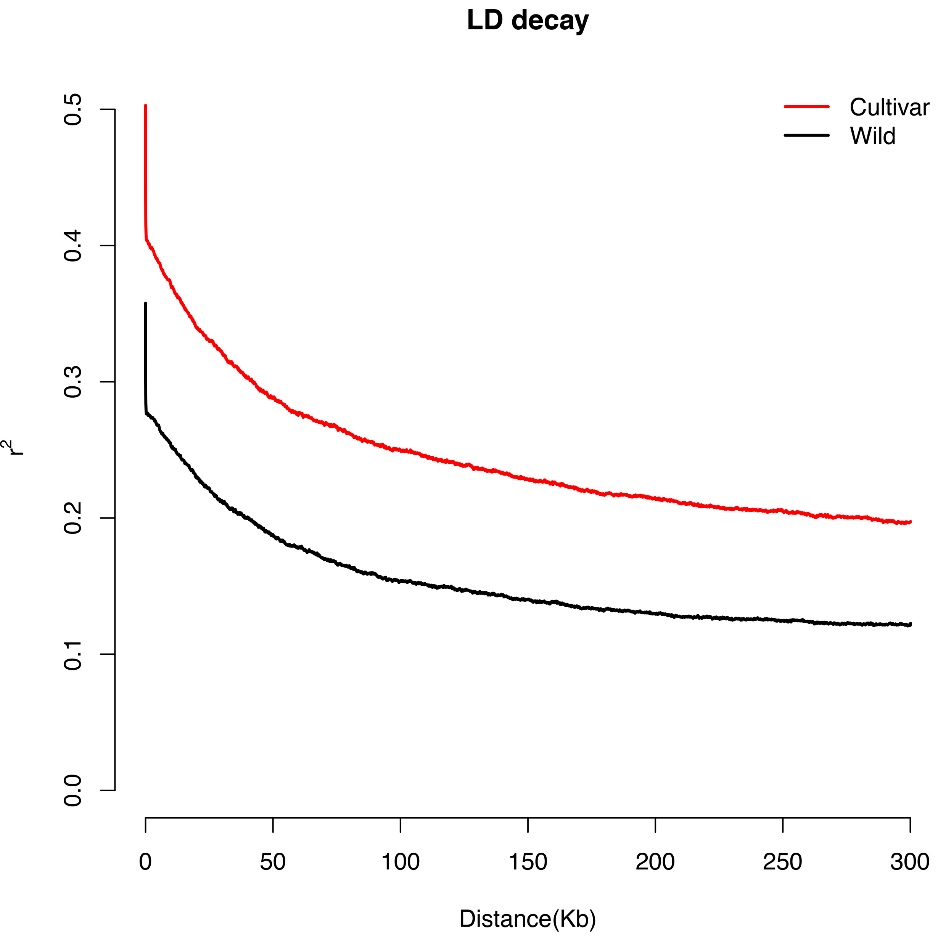


Figure S5 The decay of LD by SNPs as a function of physical distances between wild and cultivar

groups in Chinese chestnut.


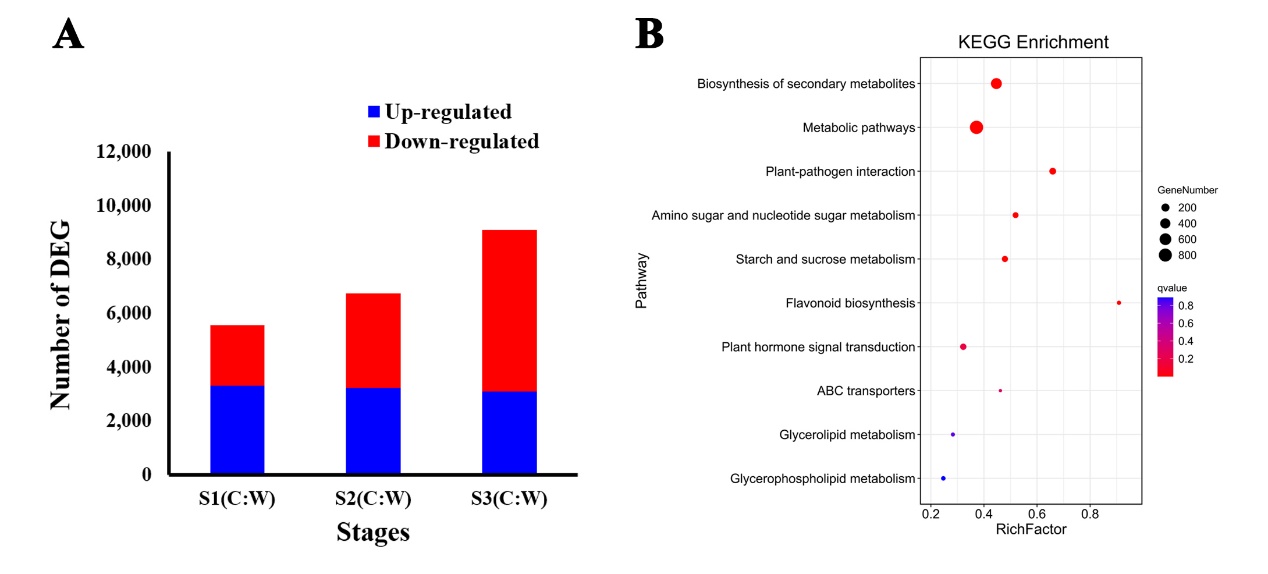


Figure S6 The number and KEGG pathway of DEGs in wild and cultivated Chinese chestnut seeds.

The number of DEG in Chinese chestnut seed germination stages. (B) The KEGG pathway of seed germination stage S3 in wild and cultivated Chinese chestnut.


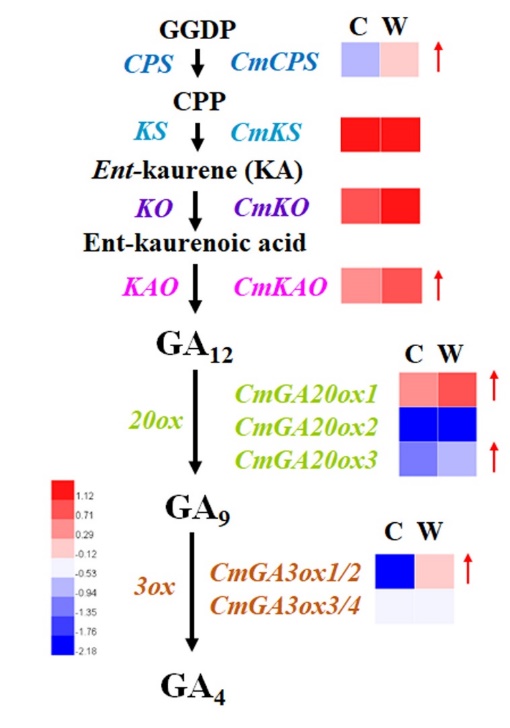


Figure S7 GA metabolic pathway for seed germination in Chinese chestnut.

A heat map was generated based on the mean expression values of the RPKM data in seed germination stage S3. C and W represent cultivar and wild seeds, respectively. The red upward arrows represent upregulated gene expression in stage S3 of wild seeds.


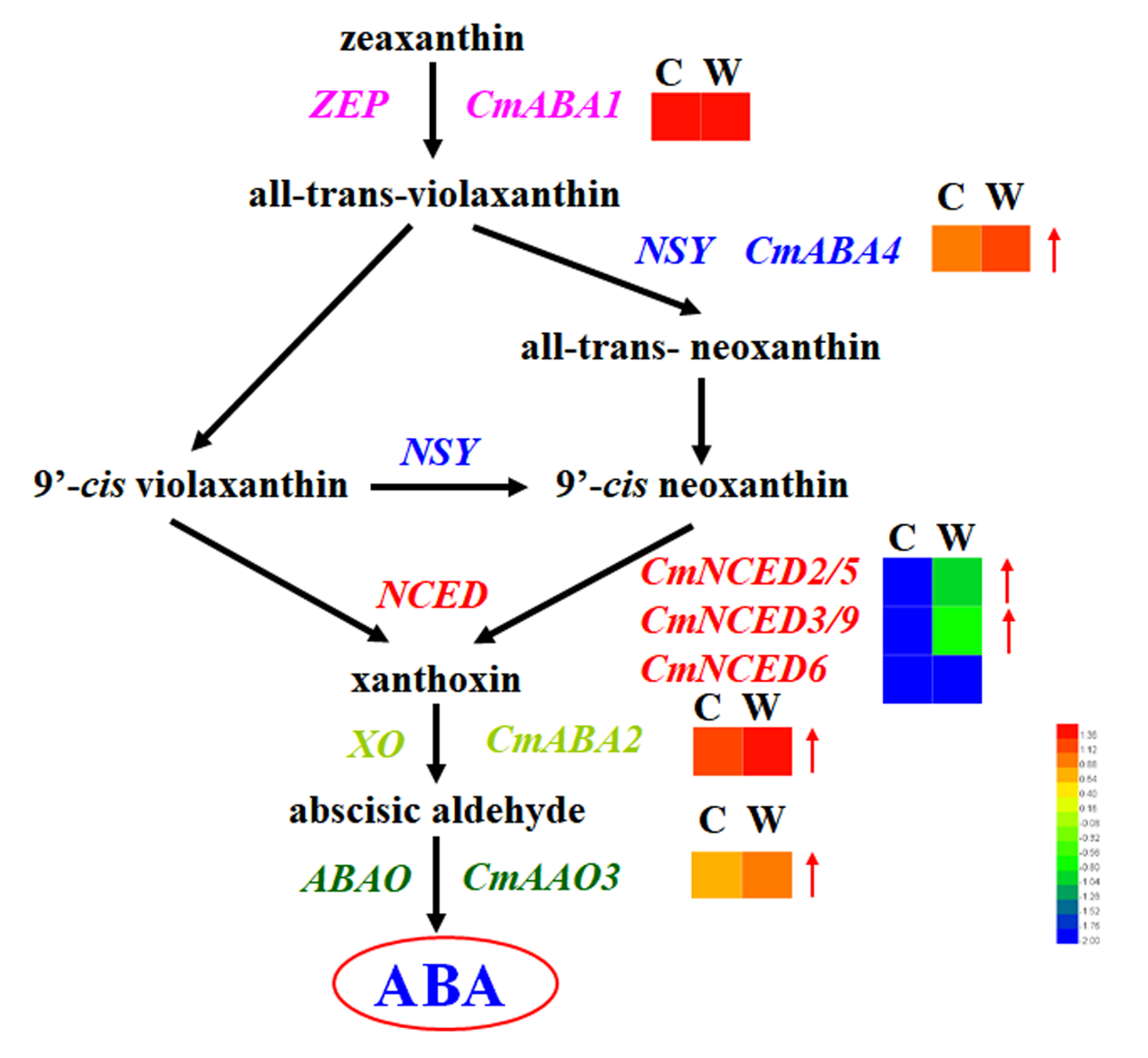


Figure S8 The ABA metabolic pathway in Chinese chestnut. A heatmap was generated based on the mean expression values of the RPKM data in seed germination stage S3. C and W represent cultivar and wild seeds, respectively. The red upward arrows represent upregulated gene expression in stage S3 of wild seeds.
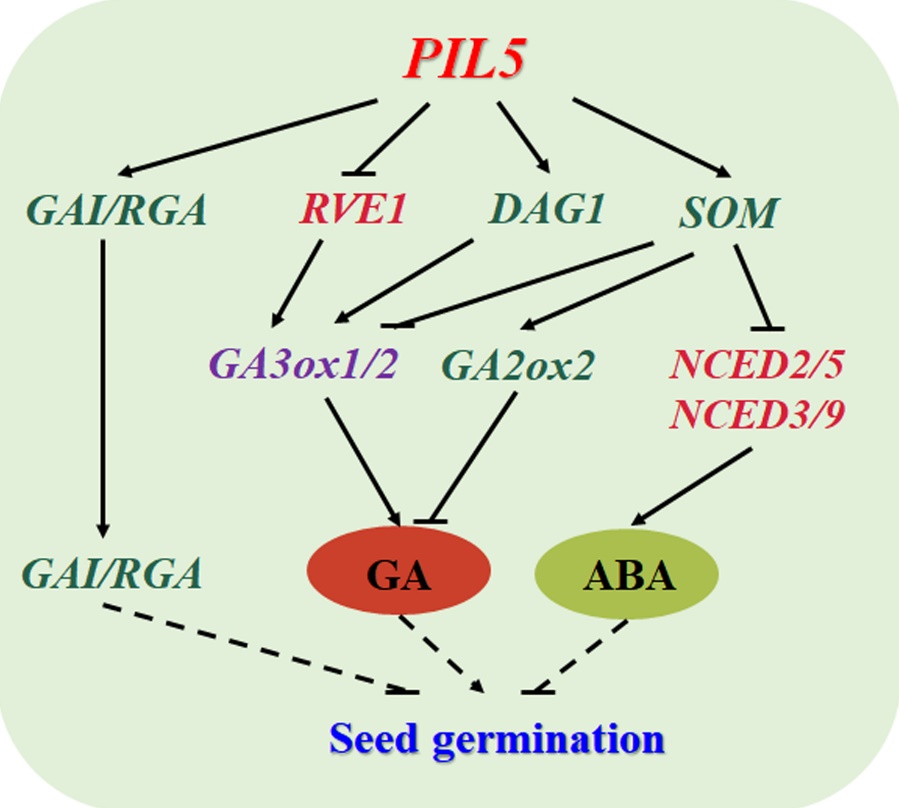


Figure S9 Model of the regulation network of Chinese chestnut seed germination.

Table S1 Statistics of Hi-C data in Chinese chestnut.

| **Statistics of Hi-C data** | | | |
| --- | --- | --- | --- |
| Number of read pairs | Clean Base(bp) | GC content (%) | Q30 (%) |
| 2,652,199 | 90,546,928,800 | 37.09 | 88.79 |
| **Statistics of mapping** | | | |
| Mapping type | | Number of reads | Ratio (%) |
| Total read pairs | | 4,574,442 | 100 |
| Mapped reads | | 8,286,211‬ | 90.57 |
| Unique mapped read pairs | | 2,652,199 | 57.97 |
| **Statistics of valid Hi-C data** | | | |
| Type | | Number of reads | Ratio (%) |
| Unique paired alignments | | 2,652,199 | 100 |
| Valid interaction pairs | | 2,340,186 | 88.24 |
| Invalid interaction Pairs | | 312,013 | 11.76 |
| Same Circularised | | 3,268 | 0.12 |
| Same Fragment Dangling Ends | | 21,960 | 0.83 |
| Same Fragment Internal | | 101,263 | 3.82 |
| Re-ligation pairs | | 90,035 | 3.39 |
| Contiguous Sequence | | 95,461 | 3.60 |
| Wrong Size | | 26 | 0.00 |

Table S2 Summary of chromosome-level assembly based on Hi-C data.

| **Chromosome** | **Number of clustered contigs** | **Length of clustered contigs (bp)** | |
| --- | --- | --- | --- |
| Chr_1 | 147 | 93,361,109 |  |
| Chr_2 | 668 | 85,524,683 |  |
| Chr_3 | 280 | 79,048,672 |  |
| Chr_4 | 183 | 66,666,324 |  |
| Chr_5 | 145 | 67,031,345 |  |
| Chr_6 | 170 | 57,051,951 |  |
| Chr_7 | 97 | 54,990,513 |  |
| Chr_8 | 83 | 54,079,050 |  |
| Chr_9 | 145 | 53,249,400 |  |
| Chr_10 | 128 | 51,514,894 |  |
| Chr_11 | 260 | 50,832,334 |  |
| Chr_12 | 172 | 41,181,582 |  |
| Total | 2,704 | 754,531,857 (97.5%) |  |

Table S3 Summary of the transcriptome determined by single molecule real-time.

| **Statistics** | **Value** |
| --- | --- |
| Sub-reads base (Gb) | 17.88 |
| Sub-reads number | 12,877,125 |
| Average sub-reads length (bp) | 1,389 |
| Number of full-length transcripts | 34,444 |
| Transcript length (bp) | 58 to 10,603 |
| Average transcript length (bp) | 1,790 |

Table S4 Genome assembly and annotation statistics for Chinese chestnut genomes.

| **Statistic** | **V2 (this study)** | **N11_1** |
| --- | --- | --- |
| **Number of scaffolds** | 886 | 112 |
| **Scaffolds N50 (bp)** | 66,666,324 | 57,343,431 |
| **Number of contigs** | 2,704 | 671 |
| **Contig N50 (bp)** | 909,265 | 2,828,629 |
| **Total size of assembled contigs** | 774.05 Mb | 688.99 Mb |
| **Genome GC content** | 37.09% | 35.11% |
| **Number of genes** | 33,991 | 33,597 |
| **Repetitive elements** | 50.25% | 53.24% |

Table S5 Genes of each chromosome in the Chinese chestnut V2 genome.

| **Chromosome** | **Repetitive Sequences (bp)** | **Repetitive Sequences (%)** | **Number of Gene** | **Average Length of CDS** |
| --- | --- | --- | --- | --- |
| Chr_1 | 43,838,327 | 46.96 | 4,499 | 1,173.60 |
| Chr_2 | 44,597,296 | 52.15 | 3,316 | 1,115.39 |
| Chr_3 | 43,693,468 | 55.27 | 2,785 | 1,133.30 |
| Chr_4 | 33,863,662 | 50.80 | 2,742 | 1,177.65 |
| Chr_5 | 32,626,792 | 48.67 | 3,146 | 1,123.55 |
| Chr_6 | 28,197,926 | 49.42 | 2,427 | 1,119.05 |
| Chr_7 | 29,410,353 | 53.48 | 2,191 | 1,125.62 |
| Chr_8 | 25,542,276 | 47.23 | 2,597 | 1,180.08 |
| Chr_9 | 26,623,362 | 50.00 | 2,397 | 1,150.17 |
| Chr_10 | 25,135,846 | 48.79 | 2,293 | 1,134.97 |
| Chr_11 | 24,666,334 | 48.52 | 2,431 | 1,101.89 |
| Chr_12 | 18,134,378 | 44.04 | 2,164 | 1,206.79 |
| Unknown | 11,001,412 | 55.84 | 1,003 | 904.96 |
| Total | 387,331,432 | 50.03 | 33,391 | 1,138.62 |

Table S6 Number of indels and genes in genomic regions.

| Region | Insertion | | Deletion | |
| --- | --- | --- | --- | --- |
|  | SVs Count | Gene | SVs Count | Gene |
| intergenic | 31,744 | 19,172 | 48,154 | 21,559 |
| intronic | 7,679 | 4,359 | 12,238 | 6,180 |
| upstream | 2,616 | 2,415 | 4,255 | 3,849 |
| downstream | 2,082 | 1,981 | 3,228 | 3,062 |
| exonic | 441 | 426 | 804 | 780 |
| splicing | 8 | 9 | 25 | 25 |
| upstream;downstream | 95 | 186 | 105 | 197 |
| 5’UTR | 3 | 3 | 0 | 0 |
